# Risks of SARS-CoV-2 Infection and Immune Response to COVID-19 Vaccines in Patients With Inflammatory Bowel Disease: Current Evidence

**DOI:** 10.3389/fimmu.2022.933774

**Published:** 2022-06-23

**Authors:** Susanna Esposito, Caterina Caminiti, Rosanna Giordano, Alberto Argentiero, Greta Ramundo, Nicola Principi

**Affiliations:** ^1^ Pediatric Clinic, Department of Medicine and Surgery, University of Parma, Parma, Italy; ^2^ Research and Innovation Unit, University Hospital of Parma, Parma, Italy; ^3^ Department of Public Health, AUSL Parma, Parma, Italy; ^4^ Università degli Studi di Milano, Milan, Italy

**Keywords:** COVID-19, COVID-19 vaccine, Crohn’s disease, inflammatory bowel diseases, SARS-CoV-2, ulcerative colitis

## Abstract

Inflammatory bowel diseases (IBD), including Crohn’s disease, ulcerative colitis, and unclassified inflammatory bowel disease, are a group of chronic, immune mediated conditions that are presumed to occur in genetically susceptible individuals because of a dysregulated intestinal immune response to environmental factors. IBD patients can be considered subjects with an aberrant immune response that makes them at increased risk of infections, particularly those due to opportunistic pathogens. In many cases this risk is significantly increased by the therapy they receive. Aim of this narrative review is to describe the impact of SARS-CoV-2 infection and the immunogenicity of COVID-19 vaccines in patients with IBD. Available data indicate that patients with IBD do not have an increased susceptibility to infection with SARS-CoV-2 and that, if infected, in the majority of the cases they must not modify the therapy in place because this does not negatively affect the COVID-19 course. Only corticosteroids should be reduced or suspended due to the risk of causing severe forms. Furthermore, COVID-19 seems to modify the course of IBD mainly due to the impact on intestinal disease of the psychological factors deriving from the measures implemented to deal with the pandemic. The data relating to the immune response induced by SARS-CoV-2 or by COVID-19 vaccines can be considered much less definitive. It seems certain that the immune response to disease and vaccines is not substantially different from that seen in healthy subjects, with the exception of patients treated with anti-tumor necrosis factor alone or in combination with other immunosuppressants who showed a reduced immune response. How much, however, this problem reduces induced protection is not known. Moreover, the impact of SARS-CoV-2 variants on IBD course and immune response to SARS-CoV-2 infection and COVID-19 vaccines has not been studied and deserves attention. Further studies capable of facing and solving unanswered questions are needed in order to adequately protect IBD patients from the risks associated with SARS-CoV-2 infection.

## Background

Inflammatory bowel diseases (IBD), including Crohn’s disease (CD), ulcerative colitis (UC), and unclassified inflammatory bowel disease (IBDU), are a group of chronic, immune mediated conditions that are presumed to occur in genetically susceptible individuals because of a dysregulated intestinal immune response to environmental factors (1). Due to the presence of multiple polymorphisms of several genes strictly related to innate and adaptive immunity (2–4), IBD patients can be considered subjects with an aberrant immune response that makes them at increased risk of infections, particularly those due to opportunistic pathogens. In many cases this risk is significantly increased by the therapy they receive. The most severe IBD cases are commonly treated with immunosuppressive drugs (systemic steroids, methotrexate, thiopurines, calcineurin inhibitors, vedolizumab, anti-TNF agents, IL-12/IL-23 antibodies, and JAK inhibitors) that, although with significant differences, further reduce immune defense efficiency and favor infections (5). A recent study carried out in 6,914 patients has shown that the long-life prevalence of clinically significant infections in these subjects is 3% and it increases to 5% in immunosuppressed subjects. Impact on quality of life of infections can be devastating as in most of the cases they add further medical visits, therapy and hospitalizations to a disease that can be per se very challenging and lead to death in about 3% of the cases (6). To prevent infectious disease development, careful monitoring of IBD patients together with a rational use of presently available vaccines are recommended. Generally, inactivated vaccines are given to all IBD subjects although immune response can be reduced in patients receiving immunosuppressive therapy (7–9). Live vaccines are generally prescribed only to IBD patients that are not treated with immunosuppressive drugs (8, 9).

After its emergence in December 2019, the Coronavirus infectious disease-19 (COVID-19) due to the severe acute respiratory syndrome-coronavirus 2 (SARS-CoV-2) has proven to be a dramatic health problem worldwide. As of 6 March 2022, over 433 million confirmed COVID-19 cases and over 5.9 million deaths have been reported globally (10). This threat has led to an extraordinary vaccine development response. In few months several COVID-19 vaccines prepared with innovative technologies were authorized for use in adults and, more recently, even for children older than 5 years of age showing high efficacy and safety in the general population. Presently, three vaccines are licensed or authorized for emergency use in the USA, two mRNA vaccines (PfizerBioNTech vaccine and Moderna vaccine) and one adenovirus vectored vaccine (Johnson and Johnson) (11). In Europe a second adenovirus vectored vaccine (AstraZeneca) and a protein-based vaccine (Novavax) have been authorized (12). Gastrointestinal problems, sometimes severe, are relatively common in patients with SARS-CoV-2 infection (13, 14). However, what is the impact of the infection on patients with already known gastrointestinal disease such as IBD is not known. In addition, the clinical relevance of COVID-19 and the true efficacy of COVID-19 vaccines in IBD patients are not precisely defined. It is debated whether SARS-CoV-2 infection and vaccines evoke in these subjects an immune response similar to that observed in healthy people so assuring a similar protection for further infections. Furthermore, it is unclear whether COVID-19 is more severe in IBD patients and whether it worsens the IBD course. Knowledge on these problems seems essential to assure the best protection of IBD patients in front of SARS-CoV-2 infection. This narrative review discusses what is presently known in this regard. The literature search was performed on the PubMed database, with a selection of English-language articles published from March 2020 to February 2022. The key search terms were “COVID-19” OR “SARS-CoV-2” OR “COVID vaccine” AND “inflammatory bowel disease” OR “Crohn’s disease” OR “ulcerative colitis”. Study titles and abstracts obtained from database searches were reviewed to identify those addressing COVID-19 disease or COVID-19 vaccine administration in IBD patients. Studies analyzing adult and pediatric patients were included. Case reports and case series were included only if strictly pertaining to these topics.

## Incidence of COVID-19 in Patients With Inflammatory Bowel Disease

There is evidence that a great number of COVID-19 patients, both adults and children, develop gastrointestinal symptoms and that, even in poorly symptomatic or asymptomatic cases, SARS-CoV-2 can be detected in the feces for prolonged periods (15, 16). The high levels of angiotensin-converting enzyme-2 (ACE-2) receptor expression in the small bowel brush border was considered the most important reason for the common gut manifestations of COVID-19 patients, although other reasons such as loss in intestinal absorption and microscopic mucosal inflammation have been supposed (17, 18). Initially, these findings led to the supposition that patients with IBD, due to aberrant immune system and the immunosuppressive effect of the drug therapy, were at higher risk of SARS-CoV-2 infection than the general healthy population (19). On the contrary, a global evaluation of the available data seems to indicate that susceptibility of IBD patients to SARS-CoV-2 infection and COVID-19 development is quite similar or even lower than that found in the general population, regardless of type of IBD, its severity and treatment. Data in this regard have been collected since the first months of pandemic both in adults and children worldwide. In China, results of a study carried out in March 2020 has shown that, among more than 20,000 adult patients followed in the 7 largest IBD centers of that country, no cases of COVID-19 were reported (20). Similarly, no COVID-19 cases were diagnosed among 318 adult patients with IBD that were prospectively followed by the Wuhan University between December 8, 2019, and March 30, 2020 (21). In Italy, in Bergamo province (i.e., one of the Italian areas with the highest incidence of COVID-19) it has been estimated that, from February 19 to March 23, 2020, a total of 46,220 patients had suffered from COVID-19, accounting for 4% of the total population (22). Despite this high prevalence, no case of diseases among 522 pediatric and adult IBD patients regularly followed was evidenced (22). In Madrid, Spain, it was evidenced that, through April 8, 2020, cumulative incidence of laboratory confirmed SARS-CoV-2 infection and risk of COVID-19 were lower in IBD patients than in the general population (4.9 case per 1,000 vs 6.2 cases per 1,000 and odds ratio [OR] 0.74, 95% confidence interval [CI] 0.70-0.77, p<0.001, respectively) (23). A study combining data collected in about 6,000 adults patients with IBD followed in France and Italy reported that the cumulative incidence of COVID-19–positive IBD patients in this cohort was 0.0025, a value quite like that observed in the general population (cumulative incidence in France and Italy in the same period was 0.0017) (24). In North Carolina, USA, the analysis of prevalence of SARS-CoV-2 infection among 14,235 individuals revealed that in the period from March 4, 2020, to April 14, 2020, positivity was 8.2% in the general population and 3.0% in IBD patients (25). Finally, a study carried out in South Korea enrolling exclusively pediatric patients definitively confirmed that children with IBD, as adults, despite the higher frequency of severe IBD cases, are not at increased risk of SARS-CoV-2 infection (26). Among the 272 children with IBD who were prospectively monitored for 2 months no cases of COVID-19 were diagnosed, although in more than 90% of the cases biologics and immunomodulators have been prescribed (26). On the other hand, the lack of any relationship between immunosuppressive therapy and increased risk of COVID-19 in IBD patient seems further demonstrated by the data collected by Khan et al. in adults (27). These authors studied 37,857 IBD patients from January 1, 2020, to May 15, 2020, and found that incidence rate of COVID-19 was 0.95 cases per 1,000 IBD. Incidence rate was not statistically different in patients treated with immunosuppressants than in those without, although slightly lower in those given these drugs (thiopurine 0.84 vs 0.96; anti-TNF 0.61 vs 1.0) (27).

The up-regulation in the blood of IBD patients of the soluble form of ACE-2 may explain why IBD patients do not have an increased susceptibility to SARS-CoV-2. Soluble ACE-2 retains the interaction site for binding to SARS-CoV-2 so acting as a competitive binding partner for the virus and reducing its infectivity (28). However, how variations in ACE-2 expression in patients with IBD, reduction in the ileum and increase in the colon impact on the risk of COVID-19 in IBD patient is not precisely defined (29).

## Clinical Course and Severity of COVID-19 in Patients With Inflammatory Bowel Disease

Whether patients with IBD who develop COVID-19 are at increased risk of adverse outcomes is not definitively ascertained. Findings reported in the Surveillance Epidemiology of Coronavirus under Research Exclusion (SECURA) IBD database have shown that age and sex-standardized mortality ratios of 3,493 IBD patients with COVID-19 were 50% higher than in the general population (30). The analysis of the same database led to the conclusion that older age, comorbidities, corticosteroid use, and male gender were associated with a higher risk of death, while therapy with biologics seemed to ensure a more favorable prognosis (30). In addition, further evidence highlighted that adverse COVID-19 outcomes could be more common in patients with more severe IBD, particularly if young patients (31). Conversely, a separate electronic health record–based study involving 31 institutions and 232 patients with IBD and COVID-19 reported that hospitalization rates and mortality of patients were not different from those evidenced in healthy matched controls (32). Moreover, Taxonera et al. reported an identical risk of death in IBD patient and the general population (OR 0.95, 95% CI: 0.84-1.06; p=0.36) (23). A lower severity of COVID-19 has been shown also by other authors (24, 33, 34). Allocca et al. found that the case fatality rate was 0% in IBD patients and 13%-15% in the general population (24). Bezzio et al. reported values of 7.6% and 13%-15% (33), and Lukin et al. of 0% and 5.9% (34), respectively. A favorable course of SARS-CoV-2 infection was reported in IBD children also. None of the 12 children with IBD (median age 14 years) described by Bosa et al. had a severe COVID-19, despite 11 of them were receiving immunomodulatory treatment (35). Four remained asymptomatic and 8 had only mild clinical manifestations. Differences in methods used to enroll and evaluate patients and controls, IBD type and severity and type and duration of drug therapy may explain different results. However, it should be highlighted that in most of the studies remained clearly evidenced that older age, obesity, severe IBD and severe underlying disease were frequently associated with severe COVID-19 course. Regarding drug therapy, it seems indisputable that corticosteroids play a relevant role in increasing the risk of a negative COVID-19 evolution, whereas other drug therapies, including biologicals, have no role in this regard or can be even associated with a reduced frequency of admission to the intensive care unit (ICU) and death. In patients receiving tofacitinib, a Janus kinase (JAK) inhibitor, occurrence of hospitalization, admission to the ICU, and severe COVID-19 was quite similar to that reported in subjects given other IBD medications (36). Indirect evidence of a potential protective effect of biological can be derived from the study by Creemers et al. (37). They compared the severity of COVID-19 in 20 IBD patients and 1,425 controls and found that rates of ICU admission (IBD 12.5% vs general population 15.7%, p=1.00), mechanical ventilation (6.3% vs 11.2%, p=1.00) and death (6.3% vs 21.8%, p=0.22) were quite similar between groups. However, none of the IBD cases with severe COVID-19 was receiving biologicals (37). Direct evidence of the different role of corticosteroids compared to biological drugs is given by Singh et al. (32). These authors analyzed the risk of severe COVID-19 in adult patients with IBD receiving different pharmacological therapy in the preceding year (37 biologics, 34 immunomodulators, 32 aminosalicylate, and 111 corticosteroids). They found that patients given immune-mediated therapy had a risk of severe COVID-19 quite similar to that shown by individuals with IBD not on immune-mediated therapy (RR 1.01; 95% CI 0.62–1.65; p=0.97) (32). On the contrary, compared to IBD patients not treated with corticosteroids, those receiving these drugs up to 3 months before the diagnosis of COVID-19 had a greater risk of severe disease (RR 1.60; 95% CI 1.01–2.57; p=0.04). Similarly, Brenner et al. analyzed the clinical course of 525 IBD cases and found that the administration of systemic corticosteroids was a significant risk factor for the development of severe COVID (OR 6.9; 95% CI 2.3-20.5) contrarily to TNF antagonists (OR 0.9; 95% CI 0.4-2.2) (38). Further evidence of a potential protective effect of TNF antagonists in IBD patient is given by the analysis of the SECURE-IBD database. Among the 2,216 patients that, as on December 7, 2022, were using anti-TNF therapy alone, 90.4% rehabilitated without hospitalization and only 10 died with a mortality rate of <1% (39). On the contrary, among the 2,035 patients treated with sulfasalazine/mesalamine only 78.3% recovered without hospitalization and 53 died with a mortality rate of 3%. Compared to other biologics, including IL-12/23 inhibitor and JAK inhibitor, anti-TNF preparations assured the highest outpatient rate of 90% (39). **Table 1** summarizes risk factors for severe COVID-19 in patients with IBD and indicate which patients with IBD and symptomatic COVID-19 may receive early treatment for reducing risk of severe desease according to the USA National Institute of Health recommendations for patients at risk (40).

**Table 1:** Patients with inflammatory bowel disease (IBD) at increased risk factors for severe COVID-19 for whom early therapy according to NIH recommendations (40) are recommended.

Risk factor	
Older age
Obesity
Severe IBD according to clinical and laboratory markers
Severe concomitant underlying disease
Corticosteroid therapy (in adults more than 20 mg/day)

Despite definitive conclusions in this regard cannot be drawn, results of some studies seem to indicate that the protective effect of the TNF antagonists in IBD patients can be ascribed to the reduction of the hyper-inflammatory responses frequently found in the most severe COVID-19 cases. In the third phase of COVID-19, over-production of pro-inflammatory cytokines including TNF-α is very common and this, together with other immune system dysregulations, is considered the main cause of the worsening of the clinical course and the increased risk of admission to the ICU and death (41). Reduction of TNF levels with the administration of effective anti-TNF preparations may significantly interfere with this phenomenon and reduce COVID-19 severity in IBD patients. On the other hand, a recent meta-analysis of 35 studies has shown that COVID-19 patients receiving anti-TNF treatment regardless of underlying diseases had a lower risk of hospitalization (OR 0.53; 95% CI 0.42-0.67) and ICU admission or death (pooled OR 0.63; 95% CI 0.41-0.96) (42). All these findings together seem to indicate that, although therapeutic approach to IBD during COVID-19 should vary according to the characteristics of the patients and IBD severity (43), in general use of all the drugs specifically prescribed for IBD, including TNF antagonist and other biologics, should be continued in case of SARS-CoV-2 infection. The only exception is represented by corticosteroids, particularly when high dosage is used. Tapering of prednisone to lower than 20 mg/day is recommended (44).

Regarding the impact of COVID-19 on IBD course, available data seem to indicate that SARS-CoV-2 infection can be associated with a worsening of gut manifestation. This seems to be mainly due to the healthcare postponement as a result of the pandemic rather than to a true impact of the virus on the gut functions. Wetwittayakhlang et al. evaluated the clinical and endoscopic activities and biomarkers of 82 IBD adult patients before and after COVID-19 infection and found that only approximately 10% had a flare of the gut disease regardless they had stopped or held medications (45). However, in most of the IBD centers, disease care was postponed or altered due to a lower healthcare priority leading to further clinical deterioration (46). Studies carried out by means of questionnaires have shown that the quality of life of IBD patients, particularly in older people, females and patients who underwent surgery, was significantly reduced during pandemic. In many cases limitations were due to mental health and emotional health problems (47).

## Immune Response of Patients With Inflammatory Bowel Disease to SARS-CoV-2 Infection

Knowledge of how in patients with IBD the diseases itself and the drug therapy they receive can condition humoral and cellular immune response to SARS-CoV-2 infection is essential to understand whether IBD and/or drug utilization are risk conditions for severe COVID-19 development and these patients are at increased risk of new SARS-CoV-2 infections. This explains why in the last months several studies have analyzed the immunity of IBD patients with COVID-19. Short-term immune response of adult patients with IBD to SARS-CoV-2 infection has been frequently found lower than that shown by healthy subjects. Several factors, including characteristics of the patient and type and severity of disease, may explain this finding. However, the major role in this regard seems to be played by the anti-TNF drugs, alone or in combination with other immunomodulators, and the shorter interval between the last drug administration and the immune system response evaluation. This does not surprise as the interference of these drugs with the immune system response to SARS-CoV-2 has been reported in all the patients receiving anti-TNF preparations, regardless of the disease for which they were prescribed (48). Regarding IBD patients, Kennedy et al. studied 6,935 patients with IBD recruited from 92 UK hospitals and measured SARS-CoV-2 infection rate and seroconversion rate in patients treated with infliximab or vedolizumab, a gut selective anti-integrin α4β7monoclonal antibody not associated with poor immune response (49). Despite rates of SARS-CoV-2 infection were similar across treatment groups, the proportion of patients seropositive for anti-SARS-CoV-2 antibody was significantly lower in infliximab-treated than in vedolizumab-treated individuals (3.4% vs 6.0%, p<0.0001). Patients given infliximab combined with other immunomodulator drugs such as thiopurine or methotrexate had a further reduction of serological response (60% vs 37%; p=0.046) (49). Wellens et al. measured the IgG and IgA production against the receptor binding domain (RBD) of the SARS-CoV-2 spike, full-length spike (S) and the nucleocapsid (N) in patients with IBD and SARS-CoV-2 infection treated with either infliximab or vedolizumab monotherapy, or infliximab/thiopurine combination therapy (50). Moreover, neutralizing SARS-CoV-2 antibodies were evaluated. Compared to healthy controls, all IBD patients had a lower IgG response to all the SARS-CoV-2 antigens. However, the greatest reduction was demonstrated in subjects receiving infliximab/thiopurine combination (p=0.00019). Regarding IgA responses and neutralizing antibody responses, they were quite similar to those evidenced in healthy subjects in patients treated with vedolizumab or infliximab monotherapy but were significantly lower in those given the infliximab/thiopurine combination [50 The poor presence of neutralizing antibodies in patients receiving combined therapy could explain why patients with IBD receiving this treatment were found to be at increased risk of severe COVID-19 than those given monotherapy (51, 52).

Regarding children, results of the few studies till now published are conflicting. Some studies seem to indicate that IBD pediatric patients are generally able to develop a significant humoral immune response against SARS-CoV-2 and that in most of the cases this is quite similar to that detected in healthy individuals of the same age, regardless of disease activity and immunosuppressive therapy. In the study by Ruan et al. seroconversion was tested in 12 subjects aged 2-17 years treated with various immunosuppressive drugs between 2.1 and 18.9 weeks (median 8.1 weeks) after initial positive SARS-CoV-2 PCR (53). The IgG antibody against the RBD of the S protein was detected in 10 (83.3%) children. All the 7 symptomatic patients seroconverted whereas no antibody was identified in the serum of 2 out of 5 asymptomatic children. A more recent study by Bosa et al. including 12 children aged 12-18 years (9 CD, 1 UC and 2 IBDU) among whom 11 were receiving immunosuppressive therapy revealed that the antibody response to SARS-CoV-2 infection was quite similar to that detected in 48 healthy children convalescent after COVID-19, matched for sex, age, time of SARS-CoV-2 infection and COVID-19 severity (27.3 ± 43.8 kAU/L vs 36.8 ± 35.3 kAU/L; p=0.451) (35). On the contrary, Dailey et al. after evaluation of humoral response of 44 IBD patients aged 11-26 years (34 with CD and 10 with UC/IBDU) who were receiving infliximab or vedolizumab alone or in combination with methotrexate concluded that humoral response of these patients approximately after 4 weeks after infection was significantly lower than that measured in otherwise healthy hospitalized pediatric patients of the same age with COVID-19 (35). No neutralizing activity was observed in 5 cases (11.4%). Several factors, including patient characteristics, type and severity of IBD, type and dosage of administered drugs, may have influenced results and may explain differences among studies.

Considering overall these results, present knowledge regarding humoral and cellular immune response to SARS-CoV-2 infection of adult and children with IBD is poor. For adults, data regarding long-term persistence of seroconversion and magnitude of immune memory are totally inadequate to draw firm conclusions and to evaluate the risk of new infections, particularly in presence of virus variants. In case of children, total number of studied pediatric patients is very small. Impact of type and severity of IBD, as well as the importance of different immunosuppressive drugs are not precisely defined. No information on persistence of humoral immunity is available. No study on cellular immunity has been performed.

## Immune Response of Patients With Inflammatory Bowel Disease to SARS-CoV-2 Vaccines

Regarding response of IBD patients to COVID-19 vaccines, a wider breadth of evidence is available, particularly regarding initial response of patients immunized with two doses of the presently available mRNA vaccines (54–70). Generally, data collected in IBD patients exposed to COVID-19 vaccines resemble those evidenced after SARS-CoV-2 infection. Age and therapy influenced magnitude of antibody production. Moreover, type of vaccine played a role. Adenovirus-vectored vaccines evoked a lower immune response than mRNA vaccines and between these, PfizerBioNTech vaccine was less effective than Moderna vaccine.

Regarding drugs, it has been repeatedly reported that patients receiving anti-TNF preparations, alone or in combination with other immunomodulators, were at higher risk of a lower humoral response, whereas this reduction was not described when different biologics or immunosuppressants were used. A series of examples can illustrate these findings. Kennedy et al. reported that after 3-10 weeks from a single dose of either the PfizerBioNTech vaccine or the AstraZeneca vaccine, seroconversion rates were lower in patients treated with infliximab than in those given vedolizumab, regardless of the vaccine used (62). However, a more detailed analysis revealed that antibody responses were lower in patients ≥60 years, in those using immunomodulators, and in those with CD. Those given infliximab compared to those receiving vedolizumab had a lower humoral immune response following both PfizerBioNTech vaccine (6.0 U/mL vs 28.8 U/mL; p<0.0001) and AstraZeneca vaccine (4.7 U/mL vs 13.8 U/mL; p<0.0001). Interestingly, immune response was higher in subjects with previous SARS-CoV-2 infection. When a second dose of PfizerBioNTech vaccine was given antibody concentrations significantly increased in all the patients, regardless of treatment (62). However, once again patients receiving infliximab had a lower increase than those receiving vedolizumab (infliximab 158 U/mL vs 6.0 U/mL, p<0.0001; vedolizumab 562 U/mL vs 28.8 U/mL, p=0.018). This finding led the authors to conclude that a single dose of COVID-19 is not enough to protect IBD patients receiving infliximab and that IBD patients, even in the presence of TNF blockade, should be prioritized for optimally timed second vaccine doses (62).

In a study enrolling 1,909 IBD adult patients, differences among vaccines were highlighted (58). It was shown that, within 90 days of the second vaccine dose, patients given mRNA vaccines had antibodies against RBD S protein in 96% of the cases, compared to 81% of those receiving the adenovirus vectored vaccine. Moderna vaccine was associated with a better immune response than Pfizer vaccine. The analysis of the factors that could have influenced the immune response revealed that, together with the vaccine preparations, also the time from the second dose and age were associated with increased risk of poor immune response. A 45% decrease in antibody level per month and a 12% decrease in antibody level per decade was demonstrated. Regarding drugs, it was evidenced that combination therapy with anti-TNF and 6MP, azathioprine, or methotrexate could condition a lack of antibody response (OR 4.2, 95% CI 2.4–7.3). The opposite was shown for mesalamine or sulfasalazine (OR 0.3, 95% CI 0.1–0.8) and ustekinumab (OR 0.2, 95% CI 0.05–0.8). Strictly dependent on age, type of given vaccine and drug exposure is also the persistence of humoral response after the second vaccine dose. Alexander et al. compared serum anti-SARS-CoV-2 S protein antibody concentrations in healthy adults (n=88) and in IBD patients (n=287) (71). All the enrolled individuals had not been previously infected by SARS-CoV-2 and had received two doses of COVID-19 vaccines (either AstraZeneca, Pfizer–BioNTech, or Moderna) 6–12 weeks apart. Blood samples for antibody titer evaluation were collected 53-92 days after the second dose. Antibody levels were found significantly lower in older patients (geometric mean ratio [GMR] 0.79, 95% CI 0.72–0.87, p<0.0001) and higher in those given mRNA vaccines (GMR 3.68, 95% CI 2.80–4.84, p<0.0001 vs Astra Zeneca vaccine) (71). Regarding role of drugs, it was shown that receiving infliximab and tofacitinib were independently associated with poor antibody response (GMR 0.12, 95% CI 0.08-017, p<0.0001 and GMR 0.43, 95% CI 0.23-0.81, p=0.0095) where no influence was exerted by ustekinumab (GMR 0.69, 95% CI 0.41–1.19, p=0.18), vedolizumab (GMR 1.16, 95% CI 0.74–1.83, p=0.51), and thiopurines (GMR 0.89, 95% CI 0.64–1.24, p=0.50) (71).

Similar findings were reported by Vollemberg et al. who extended the evaluation of the long-term persistence of anti-SARS-CoV-2 S protein antibody concentrations up to 6 months after the second vaccination and, in addition, measured the potential neutralizing capacity (VNT) of the antibodies (72). A total of 95 IBD patients and 38 healthy controls were enrolled. They received either the Pfizer-BioNTech or the Moderna vaccine. Compared to controls, patients showed lower neutralizing capacity and lower IgG anti-S concentrations both 3 and 6 months after the second vaccine dose (3 months: VNT p=0.002; 6 months: VNT p=0.062). Subjects receiving anti-TNF preparations had the lowest immune response. These and other similar findings clearly indicate that anti SARS-CoV-2 antibody concentrations despite declining even in healthy subjects, tend be very low after 6 months from the second dose in IBD patients, particularly when they are treated with anti-TNF preparations (59, 60, 73). This means that, even more than in healthy subjects, a booster dose is essential to assure protection against COVID-19 in these patients and long-term efficacy of this dose should be adequately monitored to evidence the need for periodical vaccine administrations. This conclusion seems supported by the evidence that the cellular immune response of IBD patients to COVID-19 vaccines is not precisely defined and results of studied concerning the impact of drugs used to treat IBD are not always in agreement with those relating to the humoral response. Reuken et al. reported that, after a first dose of a mRNA vaccine, T-cell responses of 28 IBD adult patients, even when a significant humoral response was not detected, was comparable to those seen in healthy subjects without influence by the drug used (63). In patients receiving a second dose induction of SARS-CoV2-reactive T-cells remained significant. In a bigger study, the good cellular response of IBD patients after COVID-19 vaccine was confirmed. In a group of IBD patients it was shown that 80% of subjects mounted a detectable T-cell response without differences between those given infliximab (n=225) and those treated with vedolizumab (n=76) (63). Different results were reported by Li et al., who studied 303 subjects immunized in 95% of the case with a mRNA vaccine, quantifying the breadth and depth of the T-cell clonal response after 8 weeks after the dose 2 administration (64). Reduced T-cell clonal depth was evidenced in older people, males, and subjects receiving immune-modifying therapy. Differently, cellular response was conserved in IBD patients treated with biologics targeting IL-12/23 and integrins, and, contrarily to the expectations, was augmented in cases given anti-TNF therapy (63). A separate discussion is needed for children as very few data, generally limited to older subjects are available. Some indications can be derived from the study by Dailey et al., despite this study enrolled mainly young adults rather than children (54). The antibody response to natural infection vs the response to immunization was tested in 33 IBD subjects aged 16-27 years old (mean age, 21 years) among whom 28 had received a mRNA vaccine (21 Pfizer-BioNTech and 7 Moderna) and 5 the Adenovirus vector vaccine (AVV, Johnson & Johnson). Antibodies against SARS-CoV-2 were measured 3.3 weeks (range, 1–10 weeks) after the second dose vaccination for the mRNA vaccines and after 3.1 weeks (range, 1.6–3.6 weeks) after the AVV vaccine. Anti-S protein IgG antibody titers were found several times higher following vaccination in comparison with natural infection in the IBD subgroup, despite slightly lower in patients with infliximab monotherapy and infliximab along with methotrexate. Production of neutralizing antibody was evidenced even when they were tested against virus variants (65). Poor impact of variants on immune response to mRNA vaccines in IBD patients seems confirmed by the evidence that in subjects receiving antimetabolite therapy (azathioprine or methotrexate), TNF inhibitors and/or other biologic treatment (anti-integrin or anti-p40) for up to 6 months after completing two-dose COVID-19 mRNA vaccination a favorable profile of vaccine-induced T cell responses was found, despite they were infected by the omicron variant (74).

## Efficacy of COVID-19 Vaccines in Patients With Inflammatory Bowel Disease (IBD)

Available data regarding efficacy of COVID-19 vaccines in IBD patients are few. A study carried out in Israel involving 12,231 patients and more than 36,000 healthy controls immunized with 2 doses of PfizerBioNTech vaccine revealed that the vaccine was very effective as infection rate was only 0.1%, regardless of the therapy (54). Slightly lower efficacy was reported in a study carried out in the USA in which 14,697 IBD patients were enrolled. Full vaccination status with one of the mRNA vaccines was found to induce protection from SARS-CoV-2 infection in 80.4% of the cases, a value significantly lower than that reported in healthy subjects (66). Data on long-term protection in IBD patients receiving the booster dose are not available. Moreover, it must be highlighted that data on protection have been collected in patients infected by the wild SARS-CoV-2 and that it is not known the role of the virus variants in modifying vaccine efficacy.

## Conclusions

Available data indicate that patients with IBD do not have an increased susceptibility to infection with SARS-CoV-2 and that, if infected, in the majority of the cases they must not modify the therapy in place because this does not negatively affect the COVID-19 course. Only corticosteroids should be reduced or suspended due to the risk of causing severe forms. Furthermore, COVID-19 seems to modify the course of IBD mainly due to the impact on intestinal disease of the psychological factors deriving from the measures implemented to deal with the pandemic, just as it has been for other chronic diseases whose assistance has inevitably been reduced. The data relating to the immune response induced by SARS-CoV-2 or by COVID-19 vaccines can be considered much less definitive. It seems certain that the immune response to disease and vaccines is not substantially different from that seen in healthy subjects, with the exception of patients treated with anti-TNF alone or in combination with other immunosuppressants who showed a reduced immune response. How much, however, this problem reduces induced protection is not known. Furthermore, it is not clear what is the effective duration of the induced protection, and which is the most suitable vaccine schedule to allow the maximum protection of IBD patients. Moreover, the role of vaccines in IBD patients infected with variants of SARS-CoV-2 has been very poorly studied. Finally, little or nothing is known about children with IBD. As reported in **Table 2**, further studies capable of facing and solving these unanswered questions are needed in order to adequately protect IBD patients from the risks associated with SARS-CoV-2 infection.

**Table 2:** Priorities for studies on COVID-19 vaccines in patients with inflammatory bowel disease (IBD).

Research area
Impact of biological drugs and other immunosuppressants on COVID-19 vaccines immunogenicity
Effective duration of protection and best vaccine schedule to allow the maximum protection
Role of vaccines in patients infected with variants of SARS-CoV-2
Immunogenicity and safety of COVID-19 vaccines in children with IBD

## Author Contributions

SE coordinated the study group and co-wrote the first draft of the manuscript. CC and RG substantially contributed to the content of the manuscript; AA and GR provided comments and suggested references; NP wrote the first draft of the manuscript. All the authors approved the final version of the manuscript.

## Conflict of Interest

SE received Speaker’s fees from GSK, Pfizer, Novartis, Sanofi Pasteur, MSD and Vifor in the past three years.

The remaining authors declare that the research was conducted in the absence of any commercial or financial relationships that could be construed as a potential conflict of interest.

## Publisher’s Note

All claims expressed in this article are solely those of the authors and do not necessarily represent those of their affiliated organizations, or those of the publisher, the editors and the reviewers. Any product that may be evaluated in this article, or claim that may be made by its manufacturer, is not guaranteed or endorsed by the publisher.
